# Chinese consumers’ psychology and behavior of the foods with nutrition claims based on AISAS model

**DOI:** 10.3389/fnut.2024.1309478

**Published:** 2024-03-01

**Authors:** Zeying Huang, Haijun Li, Jiazhang Huang

**Affiliations:** ^1^Institute of Food and Nutrition Development, Ministry of Agriculture and Rural Affairs, Beijing, China; ^2^School of Information and Intelligence Engineering, University of Sanya, Sanya, Hainan, China

**Keywords:** nutrition claim, food consumption, AISAS model, structural equation model, nutrition labeling

## Abstract

**Objective:**

We analyzed the impact of nutrition claims on Chinese consumer psychology and behavior process based on the theoretical framework of AISAS (Attention-Interest-Search-Action-Share) model.

**Design:**

To adopt questionnaires to collect gender, age, income and other basic information of adult residents and a 5-point Likert scale ranging from 1 (strongly disagree) to 5 (strongly agree) to collect data on residents’ attention to nutrition claims, interest in nutrition claims, search on nutrition claim information, purchasing behavior on food with nutrition claims, sharing information on food with nutrition claims. Then to study the relationship between the basic situation of residents and their attention, interest, search, food purchase behavior and sharing of nutrition claims by using exploratory factor analysis, reliability and validity test, structural equation modeling estimation and hypothesis testing.

**Participants:**

Chinese adults.

**Setting:**

Multi-stage stratified random sampling method was used to collect the valid online questionnaire of 630 Chinese adults from Central, North, East, South, Northwest, Southwest, and Northeast China.

**Results:**

Younger adults and those with higher household incomes exhibited heightened attention to nutrition claims. Furthermore, consumers’ attention to nutrition claims could be transformed into food information sharing through interest, information search, and food purchase. Consumers’ interest in food with nutrition claims could be transformed directly into food purchase. Consumers’ search for related information could be directly transformed into food information sharing.

**Conclusion:**

Chinese consumers’ age and household income could be included in the AISAS model for the foods with nutrition claims, and the consumers’ action and share could transform from their interest and search.

## 1 Introduction

At present, the dietary habits of Chinese adults are characterized by elevated consumption of oil and salt, accompanied by inadequate intake of whole grains, dark vegetables, fruits, milk, fish, shrimp, and beans. This dietary pattern leads to excessive sodium and saturated fatty acid intake and insufficient consumption of calcium, dietary fiber, and vitamin C (1). Therefore, the supply of food with low sodium, low fat, high calcium, rich dietary fiber, and rich vitamin C can effectively cater to residents’ requirements for a nutritious and healthy diet. The nutrition claim is a nutrition labeling that describes the positive nutritional and health properties of the foods. Marking methods and content requirements for nutrition claims on prepackaged foods have been regulated in *the National Food Safety Standard of General Rules for Nutrition Labeling of Prepackaged Foods* (GB 28050—2011) since 2011 (2). Therefore, there has been a growing number of prepackaged foods labeled with such claims in the marketplace.

Although the nutrition claim is a policy tool developed by the Chinese government for consumers to make nutritious and healthy food choices (3). However, whether and how consumers react to nutrition claims on foods is not only the basis for public authorities to evaluate nutrition policies, but also one of the indicators for manufacturers to test the market share of nutritious and healthy foods. In addition, China’s nutrition claims are introduced during the mobile Internet era when people are willing to search and share information (4). In this context, consumer responses to nutrition claims may also include the active search and sharing of nutrition claim information. However, the current research mainly focuses on consumers’ attention, interest, cognition and purchasing behavior of nutrition claims, but has not yet studied how consumers search and share nutrition claims information in the era of mobile Internet. Therefore, it is innovative to carry out research on consumers’ psychological and behavioral processes of food nutrition claims, including search and information sharing, which could understand the nutritional needs and consumption behaviors of consumers in the new era, expanding and strengthening the explanatory power of consumer behavior theories with China experience, and providing decision-making basis for governments in other countries to adjust and improve supporting measures for nutrition claims. It also provides persuasive evidence for food manufacturers to produce nutritious and healthy foods.

The remaining parts of this study are as follows: the second part summarized the research progress of the impact of nutrition claims on consumers’ psychology and behavior process; the third part proposed research hypotheses; the fourth part introduced the data sources and research methods; the fifth part implemented reliability and validity test and structural equation model estimation; the sixth part discussed the results; the final part is the conclusion and inspiration.

## 2 Literature review

**FIGURE 1:**
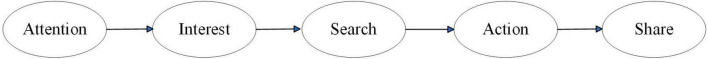
AISAS model. Source: Dentsu Inc (5).

At present, the behavior pattern of consumers in the era of mobile Internet was studied mainly with the Attention-Interest-Search-Action-Share (AISAS) model proposed by Dentsu Co., LTD in 2005 based on the Attention-Interest-Desire-Memory-Action (AIDMA) model which aims to explore consumers’ psychology and behavior in the Internet era (5). As shown in Figure 1, the AISAS model consists of attention, interest, information search, action, and experience sharing. AISAS model was widely used in online and offline marketing effectiveness evaluation of Fast Moving Consumer Goods (FMCG), durable goods and services, involving coffee, seafood, intelligent pension products, hotel accommodation, tourism service, the Augmented Reality (AR) wedding invitation app, AI-generated cultural and creative products, advertising on social networking site and organic food sold on social media. Among them, some studies extended the AISAS model according to characteristics of the products and service (6–15). For example, the social latent variables were added to the AISAS model to construct the Attention-Interest-Search-Social-Action-Share (AISSAS) model to analyze consumers’ usage behavior of the AR wedding invitation app (8). Most studies have proved that AISAS model and its extended model could effectively explain consumer psychology and behavior to products and service marketing (6–9).

There have been a number of studies on the impact of nutrition claims on consumer psychology and behavior. Through questionnaire design, scholars mainly investigated consumers’ preference, health perception, interest, willingness to pay and attention, brand loyalty, purchase decision and food choices (16–31). There also existed a few studies on the relationship between individuals’ psychology and behavior such as interest in foods with nutrition claims and purchase decision (21, 32). Studies have consistently concluded that nutrition claims had a “health halo”, which could improve consumers’ cognition of food quality, consumption confidence (17, 18, 24, 27, 28).

The existing studies on the impact of nutrition claims on consumers’ psychology and behavior were not systematic and in-depth enough to fully reveal the consumers’ reactions to nutrition claims, which failed to effectively design the promotion and marketing strategy of nutrition claims. By referring to the former studies on AISAS model and its application, we aimed to analyze the impact of nutrition claims on consumer psychology and behavior process by expanding the AISAS model and using the questionnaire survey data of Chinese consumers.

## 3 Hypotheses

**FIGURE 2:**
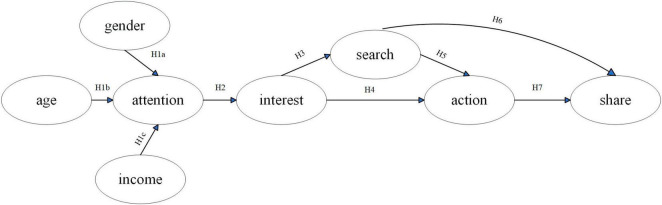
The AISAS hypothesis model. Authors’ own illustration.

The Figure 2 shows that the AISAS hypothesis model is proposed based on the framework of the AISAS model and the characteristics of foods with nutrition claims. The AISAS model does not include consumers’ socio-economic characteristics, rendering it incapable of discerning which demographic segments are more likely to pay attention to product marketing advertisements. Most studies have found that consumers who were female, younger or had higher household income paid attention to nutrition claims (33–36). It could be seen that gender, age and annual household income were the individual characteristics that significantly affected consumers’ attention to nutrition claims. Therefore, this study aims to incorporate these three individual characteristics to formulate the following research hypotheses:

H1a: Consumers’ attention to food nutrition claims is directly and negatively influenced by their gender.

H1b: Consumers’ attention to food nutrition claims is directly and negatively influenced by their age.

H1c: Consumers’ attention to food nutrition claims is directly and positively influenced by their annual household income.

The AISAS model clarifies that consumers’ interest in products is directly and positively affected by their attention to product advertising (5). Moreover, European consumers’ interest in nutrition labeling were found to be derived from attention (37, 38). It is inferred that consumers who pay attention to food nutrition claims are likely to be interested in foods with nutrition claims, so the following research hypothesis is proposed:

H2: Consumers’ interest in foods with nutrition claims is directly and positively influenced by their attention to food nutrition claims.

The AISAS model clarifies that consumers’ search for product-related information is directly and positively affected by their interest in products (5). It is also found that if nutrition labeling raised consumers’ interest, consumers would search more information about the product (39, 40). It is inferred that consumers who are interested in foods with nutrition claims are likely to search for information about those foods. Therefore, the following research hypothesis is proposed:

H3: Consumers’ search for information about foods with nutrition claims is directly and positively influenced by their interest in the food.

The AISAS model illustrates that consumers’ interest in products could only be transformed into product purchase through information search (5). Given the more affordable nature of food products featuring nutrition claims compared to items related to personal development and enjoyment (e.g., education, healthcare, entertainment, and cultural services), some consumers may be inclined to expedite their purchase decisions without extensive information search (21, 32). Consequently, the research hypothesis is as follows:

H4: Consumers’ food purchase behavior is directly and positively influenced by their interest in the foods with nutrition claims.

The AISAS model clarifies that consumers’ product purchase is directly and positively affected by their search for product-related information (5). And consumers’ search for information about foods with nutrition and health claims was found the final step that translated into foods purchasing (38). So it is inferred that consumers who search for information about foods with nutrition claims are likely to buy the foods with nutrition claims and the following research hypothesis is proposed:

H5: Consumers’ food purchase behavior is directly and positively influenced by their search for information about the foods with nutrition claims.

The AISAS model clarifies that the influence of consumers’ product information search on product information sharing goes through the food purchase process (5). In reality, some consumers without product purchase experience still share their product evaluations through we-media platforms (41). The former study proved that consumers who searched information about foods with nutrition and health claims were likely to share the relevant information (21). Therefore, the following research hypothesis is proposed:

H6: Consumers’ product information sharing is directly and positively influenced by their search for information about the foods with nutrition claims.

The AISAS model clarifies that consumers’ sharing of products is directly and positively influenced by their food purchase behavior (5). Furthermore, there was a high probability that consumers who purchased the foods with nutrition claims shared their experience (39, 42). It is inferred that consumers who have bought the foods with nutrition claims tend to share the food information. Therefore, the following research hypothesis is proposed:

H7: Consumers’ inclination to share information about foods with nutrition claims is directly and positively influenced by their purchase behavior related to these foods.

## 4 Materials and methods

### 4.1 Data collection

As indicated in Table 1, the five latent variables in the AISAS model could be measured by scale items, with respondents providing their answers on a 5-point Likert scale ranging from 1 (strongly disagree) to 5 (strongly agree). Our questionnaire (see Supplementary material) was refined based on feedback from a pre-survey involving 30 adults in Beijing, China. We utilized a paid online survey service of Wenjuanxing,^1^ a reputable online survey platform in China with a database of 6.2 million registered members of different ages across 31 provinces/autonomous regions/municipalities (referred to as ‘province’ hereafter).

**TABLE 1:** Scale items for each latent variable.

Latent variables	Scale items	Scale items code
Attention	The food nutrition claims have attracted your attention.	a1
	Manufacturers running ads highlighting food nutrition claims have attracted your attention.	a2
	Compared to other identical foods, the food with nutrition claims has attracted your attention.	a3
	You have paid attention to food nutrition claims in daily life.	a4
Interest	You have been interested in the foods with nutrition claims.	b1
	Nutrition claims have aroused your interest.	b2
	The foods with nutrition claims has made you feel better than the food without such claims.	b3
	You are eager to try the foods with nutrition claims.	b4
	You are more interested in the foods with nutrition claims than those without.	b5
Search	You have searched for the relevant information about the foods with nutrition claims before purchasing.	c1
	You have dispelled doubts about the foods with nutrition claims through some channels before purchasing.	c2
	You have verified the accuracy of the food nutrition claims through some channels before purchasing.	c3
	You have searched for more information about the foods with nutrition claims before purchasing.	c4
	You have sought other people’s evaluations of the foods with nutrition claims before purchasing.	c5
Action	You have purchased the foods with nutrition claims.	d1
	You have purchased the foods with nutrition claims for your friends and family members.	d2
	You have made the purchase plan for the foods with nutrition claims.	d3
	You often buy the foods with nutrition claims.	d4
Share	You have shared the consumption experience of the foods with nutrition claims with your friends and family members.	e1
	You have given a positive evaluation for the foods with nutrition claims.	e2
	You have shared your positive experience of the foods with nutrition claims on the Internet.	e3
	You have recommended the foods with nutrition claims to strangers.	e4
	You have shared information about the foods with nutrition claims on your social media, such as WeChat Moments or Weibo.	e5

The scale items on consumers’ attention, interest and search referred to Fannani and Najib (10) while the scale items on consumers’ action and share referred to Javed et al. (13).

This study adopted multi-stage stratified random sampling method. Due to the fact that people in Northeast China, North China, Northwest China, East China, Central China, Southwest China, and South China have different eating habits, China was divided into the above seven regions in the first stage, and then one province was randomly selected from these regions (43). The seven provinces were Jilin, Inner Mongolia, Shaanxi, Shandong, Henan, Sichuan, and Guangdong. Finally, according to the minimal number of representative, random samples (*N* = 600) in China was determined with an allowable error of 4% and a confidence level of 95%, at least 86 samples collected from each selected province on average. Therefore, we commissioned Wenjuanxing to collect target sample size from its member database. From September 8th to September 22nd, 2022, Wenjuanxing emailed the questionnaire link to 130 adults randomly selected from each province, and about 88.13% participated in the online survey. Before data collection, informed written consent was obtained from all participants. Eight Chinese Yuan as cash incentives were offered to each respondent if their responses were careful and complete. Finally, after data validity was checked, 630 valid samples were used for analysis.

### 4.2 Methods

Although the linear regression model could be used to analyze consumer behavior based on AISAS model (12), most previous studies adopted the structural equation model to analyze the internal relationship between consumers’ attention, interest, search, action and sharing (11, 15). Therefore, we intended to use this method. Structural equation modeling allows the creation of observable variables per construct, which does not require split analysis and yields valid and clear inferences (44). Thus, the results of the relationships among variables were reliable and neutral (45). In addition, structural equation modeling is capable of scrutinizing complex correlations and a range of hypotheses by immediately incorporating mean structures and group estimation (46).

According to the influence paths in Figure 2, the hypotheses proposed above were made out by the structural equation modeling which consists of a structural model and a measurement model.


(1) η=B⁢η+Γ⁢ξ+ζ

The formula above is a structural model. η are the latent variables of the AISAS model. *B* are the relationships between latent variables. ξ are exogenous variables such as gender, age and annual household income. Γ is the influence of exogenous variables on individuals’ attention to nutrition claims. ζ is the residual term.


(2) $$Y=\hat{y}\,\eta +\epsilon$$

The formula above is a measurement model. *Y* are explicit variables. $\hat{y}$ is the correlation coefficient matrix of latent variables (*i.e.*, attention, interest, search, action, share) and their explicit variables. ϵ is measurement error.

Then the built model estimation was conducted in two steps. Firstly, the reliability and validity test was performed to evaluate the stability and consistency of measured items in the measurement model with the SPSS Statistics 24.0 and AMOS Statistics 26.0. Secondly, the evaluation of goodness-of-fit indices for the proposed structural equation modeling and tests of hypotheses were made by means of moment structure analysis with Stata Statistics17.0 to obtain the direct effect, indirect effect and total effect among latent variables.

## 5 Results

### 5.1 Descriptive statistics

As shown in Table 2, the distribution of samples’ gender and residence closely resembled the *Main data of the 7th National Population Census in 2020* (47). This validates the representativeness of the overall sample despite of low proportion of the population aged 60 and above and with junior high school education and below.

**TABLE 2:** Samples’ characteristics.

Characteristics	Items	Samples	Percentage (%)	The 2020 Population Census Data^b^ (%)
Gender	Male	315	50	51.24
	Female	315	50	48.76
Age	18∼59 years old	616	97.78	86.30
	60 years old and above	14	2.22	13.70
Education level	Junior school or below	27	4.28	17.6
	Senior school	428	67.94	55.03
	Junior college	154	24.44	24.61
	Postgraduate	21	3.33	2.76
Residence	Urban area	378	60	63.89
	Rural area	252	40	36.11
Annual household disposable income (Chinese Yuan^a^)	<10,000	31	4.92	–
	10,000∼49,999	114	18.10	–
	50,000∼99,999	123	19.52	–
	100,000∼149,999	143	22.70	–
	150,000∼199,999	116	18.41	–
	≥200,000	103	16.35	–

Authors’ own calculation. *^a^*One US dollar equals 6.980 Chinese Yuan, and one Euro equals 6.900 Chinese Yuan from September 8th to September 22th, 2022. *^b^*Annual household disposable income was not measured in the *Population Census in 2020*.

Based on the information provided in the Table 3, several conclusions can be drawn regarding respondents’ perceptions and behaviors in relation to the marketing of foods with nutrition claims.

**TABLE 3:** Description of latent variables and summary statistics.

Latent variables	Scale items code	Centralized and discrete statistics				Strongly disagree		Disagree		Neither agree nor disagree		Agree		Strongly agree	
		Mean	Std dev	Skewness	Kurtosis	*N*	%	*N*	%	*N*	%	*N*	%	*N*	%
Attention	a1	4.00	0.67	−0.53	3.77	0	0	17	2.70	92	14.60	395	62.70	126	20.00
	a2	4.02	0.76	−0.74	3.85	2	0.32	25	3.97	91	14.44	353	56.03	159	25.24
	a3	4.17	0.72	−0.77	4.01	1	0.16	16	2.54	65	10.32	344	54.60	204	32.38
	a4	3.98	0.79	−0.63	3.33	1	0.16	30	4.76	106	16.83	338	53.65	155	24.60
	a5	3.91	0.80	−0.78	3.82	3	0.48	38	6.03	98	15.56	363	57.62	128	20.32
Interest	b1	4.06	0.78	−0.65	3.34	1	0.16	23	3.65	98	15.56	324	51.43	184	29.21
	b2	4.06	0.76	−0.68	3.42	0	0	27	4.29	84	13.33	341	54.13	178	28.25
	b3	4.20	0.78	−0.97	4.06	2	0.32	22	3.49	64	10.16	300	47.62	242	38.41
	b4	4.08	0.76	−0.78	4.07	3	0.48	18	2.86	87	13.81	340	53.97	182	28.89
	b5	3.98	0.79	−0.73	3.67	2	0.32	31	4.92	96	15.24	348	55.24	153	24.29
Search	c1	4.07	0.78	−0.86	4.20	4	0.63	21	3.33	85	13.49	336	53.33	184	29.21
	c2	3.81	0.82	−0.49	2.97	1	0.16	45	7.14	141	22.38	329	52.22	114	18.10
	c3	3.94	0.87	−0.57	2.89	2	0.32	39	6.19	127	20.16	289	45.87	173	27.46
	c4	3.86	0.94	−0.86	3.61	13	2.06	46	7.30	110	17.46	309	49.05	152	24.13
	c5	3.62	0.98	−0.55	2.84	15	2.38	75	11.90	152	24.13	280	44.44	108	17.14
Action	d1	4.02	0.70	−0.67	4.31	2	0.32	15	2.38	90	14.29	385	61.11	138	21.90
	d2	4.04	0.85	−0.88	3.89	6	0.95	27	4.29	96	15.24	305	48.41	196	31.11
	d3	4.02	0.90	−1.06	4.31	12	1.90	29	4.60	88	13.97	305	48.41	196	31.11
	d4	3.87	0.84	−0.75	3.80	8	1.27	31	4.92	130	20.63	328	52.06	133	21.11
Share	e1	3.91	0.83	−1.09	4.90	11	1.75	30	4.76	88	13.97	374	59.37	127	20.16
	e2	3.74	0.93	−0.53	2.99	10	1.59	49	7.78	167	26.51	272	43.17	132	20.95
	e3	3.60	1.05	−0.55	2.67	21	3.33	84	13.33	142	22.54	262	41.59	121	19.21
	e4	2.93	1.12	−0.05	2.22	74	11.75	150	23.81	197	31.27	162	25.71	47	7.46
	e5	3.56	1.04	−0.64	3.00	31	4.92	62	9.84	165	26.19	266	42.22	106	16.83

Authors’ own calculation.

As for attention, the majority of respondents (62.70%) agreed that the food nutrition claims attracted their attention. There existed variability, indicated by the standard deviation of 0.67. As for interest, there was significant interest from respondents in foods with nutrition claims, with around 86.03% of them agreeing that these foods made them feel better than those without such claims. Variability in responses existed, suggested by the standard deviation of 0.78. As for search, respondents appeared to search for information about foods with nutrition claims, but there was significant variation in the level of search, indicated by the standard deviation of 0.87. A few people (3.33%) indicated that they completely agreed with actively seeking information. As for action, most respondents (83.01%) agreed or strongly agreed that they bought foods with nutrition claims, but there was variability, suggested by the standard deviation of 0.70. As for share, there was a variety of attitudes regarding sharing information about foods with nutrition claims, and the lowest percentage was associated with recommending these foods to strangers (20.16%). There was significant variation in the level of sharing, indicated by the standard deviation of 0.83.

### 5.2 Exploratory factor analysis

As was shown in Table 4, the Kaiser-Meyer-Olkin (KMO) statistics of scale items and overall KMO statistics were above 0.7 and the approximate chi-square value of Barlett’s sphericity test of five latent variables and overall Bartlett value were statistically significant. It means that the samples were suitable for exploratory factor analysis. The results of factor loading after rotation indicated that eigenvalue of 5 factors were greater than 1, and the cumulative contribution rate was more than 80%.

**TABLE 4:** Factor loading matrix after rotation.

Scale items code	Factor 1	Factor 2	Factor 3	Factor 4	Factor 5	KMO	Bartlett value
a1	0.693	−0.104	0.528	0.508	0.564	0.932	459.595***
a2	0.602	−0.209	0.473	−0.158	0.065	0.941	
a3	0.706	−0.126	0.512	0.413	−0.058	0.940	
a4	0.602	−0.070	0.458	0.343	0.137	0.959	
a5	0.558	−0.155	0.228	0.202	0.678	0.938	
b1	0.727	−0.173	0.202	−0.061	−0.209	0.944	658.631***
b2	0.697	−0.197	0.183	−0.131	−0.213	0.949	
b3	0.582	−0.232	0.111	0.458	−0.217	0.939	
b4	0.510	−0.132	0.215	−0.068	−0.074	0.954	
b5	0.615	−0.161	0.161	−0.078	−0.078	0.957	
c1	0.600	0.535	0.053	0.531	−0.221	0.946	621.254***
c2	0.777	0.440	0.163	0.753	0.185	0.918	
c3	0.672	0.642	0.142	0.349	−0.133	0.894	
c4	0.505	0.507	0.216	0.531	−0.179	0.925	
c5	0.672	0.315	0.115	0.332	0.216	0.924	
d1	0.528	−0.105	0.310	0.258	0.095	0.958	1637.266***
d2	0.615	−0.184	−0.143	0.209	−0.061	0.759	
d3	0.616	−0.158	−0.259	0.302	−0.101	0.757	
d4	0.532	−0.100	−0.084	−0.113	0.265	0.955	
e1	0.608	0.733	−0.160	−0.180	−0.185	0.949	920.893***
e2	0.623	0.612	−0.122	−0.350	0.015	0.944	
e3	0.512	0.677	−0.103	−0.273	−0.005	0.929	
e4	0.595	0.704	−0.193	−0.270	0.166	0.917	
e5	0.529	0.636	−0.057	−0.290	−0.074	0.925	
Cumulative variance%	43.612	54.941	66.780	71.864	84.206	91.562	

Authors’ own calculation; Overall Bartlett’s Test of Sphericity: χ2 = 5986.933, ***p < 0.01; Overall KMO was 0.915.

### 5.3 Reliability and validity test

As seen from Table 5, the corrected item-total correlation coefficients of all scale items were greater than 0.5, indicating a strong correlation between scale items. All the Cronbach’s α were above 0.8 if any item was deleted. However, the Cronbach’s α was highest (0.906) if a5 was deleted.

**TABLE 5:** Corrected item-total correlation results.

Scale items code	Corrected item-total correlation	Cronbach’s α if item deleted
a1	0.660	0.818
a2	0.556	0.846
a3	0.566	0.801
a4	0.582	0.811
a5	0.577	0.906
b1	0.568	0.811
b2	0.649	0.825
b3	0.593	0.840
b4	0.588	0.882
b5	0.535	0.857
c1	0.645	0.842
c2	0.563	0.813
c3	0.683	0.855
c4	0.556	0.845
c5	0.568	0.876
d1	0.556	0.806
d2	0.592	0.801
d3	0.578	0.879
d4	0.637	0.856
e1	0.574	0.804
e2	0.583	0.812
e3	0.601	0.874
e4	0.591	0.852
e5	0.574	0.845

Authors’ own calculation.

Table 6 shows that Cronbach’s α of each latent variable was greater than 0.7 when deleting a5, indicating that the scale reliability was good.

**TABLE 6:** Reliability test results.

Latent variables	Scale items code	Cronbach’s α
Attention	a1	0.712
	a2	
	a3	
	a4	
Interest	b1	0.757
	b2	
	b3	
	b4	
	b5	
Search	c1	0.742
	c2	
	c3	
	c4	
	c5	
Action	d1	0.806
	d2	
	d3	
	d4	
Share	e1	0.804
	e2	
	e3	
	e4	
	e5	

Authors’ own calculation.

As shown in Table 7, Average Variance Extracted (AVE) of all constructs were more than 0.7. It means that constructs had high reliability and convergence validity. The discriminant validity among constructs were assessed with Maximum of shared squared variance (MSV) and Average of shared squared variance (ASV), and Table 8 showed that the scale could distinguish different dimensions or concepts to a high degree.

**TABLE 7:** Correlation coefficient matrix.

Latent variables	Attention	Interest	Search	Action	Share
Attention	[0.972]				
Interest	0.968***	[0.759]			
Search	0.664***	0.648***	[0.792]		
Action	0.677***	0.500***	0.387***	[0.822]	
Share	0.695***	0.647***	0.752***	0.752***	[0.879]

Authors’ own calculation; ***p < 0.01. AVE of all latent variables in parentheses.

**TABLE 8:** Discriminative validity test.

Latent variables	CR	MSV	ASV
Attention	0.924	0.386	0.352
Interest	0.919	0.309	0.257
Search	0.967	0.275	0.223
Action	0.971	0.186	0.189
Share	0.902	0.428	0.276

Authors’ own calculation.

### 5.4 Structural equation modeling estimation and hypothesis testing

Table 9 displays the goodness-of-fit indices for the initial and modified models. None of the fitting indexes of the initial model reached the threshold value. The initial model was modified by adding the residual correlation path between explicit variables (i.e., d2 and d3). The fitting index values such as Standardized Residual Mean Root (SRMR), Root Mean Square Error of Approximation (RMSEA), Tucker-Lewis Index (TLI), Comparative Fit Index (CFI), Parsimony Goodness-of-Fit Index (PGFI), Parsimony Normed Fit Index (PNFI) and Parsimony Comparative Fit Index (PCFI) outperformed the respective threshold values, signifying that the model was able to fit all data satisfactorily. Likelihood ratio χ^2^_ms, χ^2^/df, Akaike Information Criterion (AIC) and Bayesian Information Criterion (BIC) in the modified model were smaller than those in the initial model.

**TABLE 9:** Structural equation modeling fitting.

Goodness-of-fit indices	Threshold value	Fitting index values in the initial model	Fitted	Fitting index values in the modified model	Fitted
Likelihood ratio χ^2^_ms	the smaller, the better	956.658	No	620.736	Yes
Chi-square (χ^2^/df)	the smaller, the better	153.22	No	114.51	Yes
SRMR	<0.05	0.083	No	0.042	Yes
RMSEA	<0.05	0.060	No	0.043	Yes
TLI	>0.9	0.874	No	0.937	Yes
CFI	>0.9	0.886	No	0.943	Yes
PGFI	>0.5	0.498	No	0.787	Yes
PNFI	>0.5	0.487	No	0.890	Yes
PCFI	>0.5	0.489	No	0.869	Yes
AIC	the smaller, the better	38936.977	No	38603.055	Yes
BIC	the smaller, the better	39283.743	No	38954.267	Yes

Authors’ own calculation.

**FIGURE 3:**
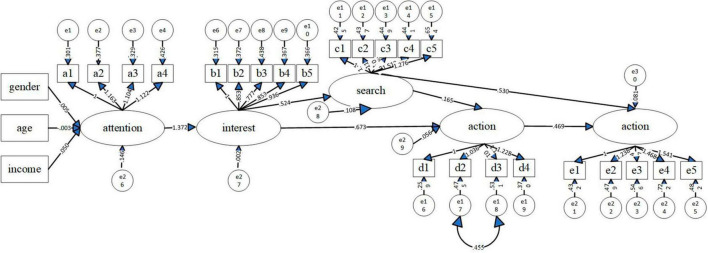
Modified structural equation modeling results. e1∼e25 are the residuals of explicit variables. e26∼e30 are the measurement errors of latent variables, Source: Authors’ own illustration.

Figure 3 shows the estimation result of the modified model. All hypotheses were supported at the statistical significance level except for the influence of gender (Table 10). To be more specific, consumers’ attention to food nutrition claims were negatively influenced by their age and positively influenced by their annual household income so H1b and H1c were supported. However, consumers’ gender took insignificant effect and H1a was not accepted. As expected, consumers’ attention posed positive influence on their interest which had positive influenced their search for information about foods with nutrition claims and food purchase behavior. Therefore, H2, H3, H4 were accepted. Furthermore, consumers’ search for the information positively affected their food purchase and sharing, supporting H5 and H6. H7 was also supported because consumers’ product information sharing was influenced by their food purchase behavior.

**TABLE 10:** Test results of the hypothesis.

Hypothesis	Hypothesized paths	Coefficient	OIM std. err	Accepted
H1a: Consumers’ attention to food nutrition claims is directly and negatively influenced by their gender.	Gender → attention	0.005	0.033	No
H1b: Consumers’ attention to food nutrition claims is directly and negatively influenced by their age.	Age → attention	−0.003**	0.001	Yes
H1c: Consumers’ attention to food nutrition claims is directly and positively influenced by their annual household income.	income → attention	0.050***	0.012	Yes
H2: Consumers’ interest in foods with nutrition claims is directly and positively influenced by their attention to food nutrition claims.	attention → interest	1.372***	0.115	Yes
H3: Consumers’ search for information about foods with nutrition claims is directly and positively influenced by their interest in the food.	interest → search	0.524***	0.055	Yes
H4: Consumers’ food purchase behavior is directly and positively influenced by their interest in the foods with nutrition claims.	interest → action	0.760***	0.057	Yes
H5: Consumers’ food purchase behavior is directly and positively influenced by their search for information about the foods with nutrition claims.	search → action	0.165**	0.067	Yes
H6: Consumers’ product information sharing is directly and positively influenced by their search for information about the foods with nutrition claims.	search → share	0.607***	0.082	Yes
H7: Consumers’ inclination to share information about foods with nutrition claims is directly and positively influenced by their purchase behavior related to these foods.	action → share	0.469***	0.071	Yes

**P < 0.05, ***p < 0.01. OIM std. err stands for observed information matrix standard error.

As shown in Table 11, among the influence paths, the effect of respondents’ attention to food nutrition claims on their interest in the food with such claims had the most significant total effect (1.372). It was followed by the influence of attention on food purchase (1.042), the influence of attention on information sharing (0.869), the influence of interest on food purchase (0.760), and the influence of attention on search for information about such foods (0.719).

**TABLE 11:** Paths effect decomposition.

Paths	Direct effect	Indirect effect	Total effect
Attention → Interest	1.372***	–	1.372***
Attention → Search	–	0.719***	0.719***
Attention → Action	–	1.042***	1.042***
Attention → Share	–	0.869***	0.869***
Interest → Search	0.524***	–	0.524***
Interest → Action	0.673***	0.087**	0.760***
Interest → Share	–	0.634***	0.634***
Search → Action	0.165**	–	0.165**
Search → Share	0.530***	0.077**	0.607***
Action → Share	0.469***	–	0.469***

**P < 0.05, ***p < 0.01.

## 6 Discussion

### 6.1 Association between Individuals’ socio-economic status and attention

Respondents’ gender did not have a direct and significant impact on their attention to food nutrition claims. It is inconsistent with previous studies that female groups were likely to pay attention to nutrition claims (33, 34). This may be due to the fact that food nutrition claims in China conveyed information that appealed to both men and women, so there were not significant gender differences in consumers’ attention to nutrition claims. In addition, respondents’ age and annual household income significantly and directly affected their attention to food nutrition claims. Specifically, the increase in age significantly decreased respondents’ attention to such claims, while the increase in annual household income raised respondents’ attention to the claims. This finding was in accordance with the studies of Klopčič et al. (36) and Vyth et al. (48) Compared with relevant studies on the improved AISAS model by incorporating latent variables (9, 13), the AISAS model in our study, which incorporated external variables including age and annual household income, represented an innovative and valuable approach for identifying the characteristics of consumers who were attentive to food nutrition claims.

### 6.2 Association between attention and interest

Respondents who paid attention to food nutrition claims increased interest in such claims, consistent with the theoretical prediction of the AISAS model (5) and relevant empirical studies that confirmed consumers’ interest was determined by their attention (11, 13). In our study, attention had the most significant influence on interest (1.372), indicating that respondents’ attention to nutrition claims was most likely to transform into interest compared to other influence paths. However, other studies argued that, of all the paths, interest had the greatest impact on search or search had the greatest impact on purchase (12, 14). This is because our study took food nutrition claims as the research object. Only when nutrition claims were paid attention could consumers become interested, and then other behaviors such as searching, buying and sharing would be carried out.

### 6.3 Association between interest and search

Respondents interested in foods with nutrition claims tended to search for information about such foods, which was consistent with the expected theoretical predictions of the AISAS model (5) and the studies’ results (14, 15). However, the difference is that the limited effect of respondents’ interest on their information search (0.524) in our study suggested a low probability of transforming interest in foods with nutrition claims into information search. Perhaps this is due to that China’s nutrition claims was authoritative and enforced by the government. Consumers who were interested in nutrition claims were generally less likely to search for information and convert it directly into buying behavior.

### 6.4 Association between interest and action

Respondents’ interest in foods with nutrition claims directly and positively influenced their purchase behavior of those foods. It indicates that emotionally driven consumers tended to consume impulsively without searching for information despite the convenience of information access in the mobile Internet era. This finding differed from the theoretical prediction of the AISAS model that represented the one-way progressive progress of interest → search → action, but was in accordance with the previous studies’ results that consumers who were interested in food nutrition claims had high probability in purchasing foods labeled with nutrition claims (21, 32). Like other studies (12, 14), this study also found that in practice, consumers did not strictly follow the theoretical model of the AISAS model, which provided Chinese experience for expanding the AISAS model.

### 6.5 Association between search and action

Respondents who searched for information about the foods with nutrition claims tended to buy food, which was consistent with the theoretical prediction of the AISAS model (5) and other studies (13, 15). However, among the many influence paths, food information search had the smallest influence on food purchasing (0.165). It implied a limited motivation for food information searchers to buy the food with nutrition claims. This is because Chinese respondents interested in the foods with nutrition claims may search for information to fully understand the food before shopping, or they may make consumption decision without searching for relevant information.

### 6.6 Association between search and share

Respondents who actively searched for information about the foods with nutrition claims were likely to share food consumption experience and information, which was not same with the theoretical prediction of the AISAS model that represents the one-way progressive progress of search → action → share (5), but was consistent with the former findings that search could have a direct impact on sharing (12, 21). It was possibly because that certain consumers who engaged in information search but lacked actual shopping experience were still willing to share the information of food with nutrition claims on we-media platforms due to the low cost of online information search.

### 6.7 Association between action and share

Respondents who bought the foods with nutrition claims tended to share food shopping experiences and information, consistent with the theoretical prediction of the AISAS model (5) and the relevant studies’ conclusion (13, 14). This confirmed that in the era of mobile Internet, many consumers had mobile devices such as cell phones. Consumers who had a good experience in buying food with nutrition claims tended to share their purchasing experience on social media to attract potential consumers to pay attention to the food with nutrition claims. In this way, a virtuous circle would be formed. Through word-of-mouth marketing, more people would be encouraged to buy nutrition and health food, and finally the nutrition and health level of the whole society could be improved.

## 7 Conclusion

This study aimed to investigate consumers’ psychology and behavior of foods with nutrition claims using questionnaire data collected from 630 adults across representative regions in China, employing the AISAS model. The findings suggested that the AISAS model with individual socio-economic characteristics and non-unidirectional progression was suitable for analyzing the reaction of Chinese adults to foods with nutrition claims. Younger adults and those with high annual household income were likely to pay attention to food nutrition claims. Consumers’ attention and interest in food nutrition claims were transformed into information search, food purchase, and experience sharing.

Our results proved that nutrition claims were able to guide specific Chinese consumer groups to make purchase decision especially share their opinion about the foods with nutrition claims, thus there were perhaps some theoretical and practical contributions of this study. Firstly, the expansion of AISAS model theory was supported by the present empirical study in China, highlighting that individuals’ socio-economic characteristics could be included in the model, and the people’s action and share could also transform from interest and search, respectively, so the updated model may have high efficiency of explanation and prediction for more forms of products’ advertising marketing. Secondly, our study was likely to provide the basis for the public sector to continue promoting nutrition claims to implement the national dietary nutrition health interventions and for enterprises to actively label nutrient-rich foods with nutrition claims which was sold in subdivided groups.

This study is subject to certain limitations which will be solved in future research. Firstly, our sample size is relatively small in comparison with China’s large population, which may restrict the generalizability of our findings to broader studies. Secondly, the model did not regard the social environment as an external factor, despite the proven role of social and economic factors in shaping individuals’ psychology and behavior. Thirdly, scale items measuring consumption process of the foods with nutrition claims with self-report questionnaires are biased toward subjectivity. However, self-report questionnaires are vulnerable to social desirability bias due to respondents tendencies to answer in a more socially acceptable way. Therefore, for accurate research results, more attempts are needed, involving a larger sample, adding social environment variables and employing indirect questioning methods such as the list experiment.

The following implications are offered: Firstly, manufacturers may focus on younger demographics and groups with higher household incomes when developing marketing strategies for food products with nutrition claims to promote the broader consumption of nutritious and healthy foods. Secondly, consumers could be encouraged to share their consumption experience and food evaluation online to guide more consumers to purchase food. Thirdly, public sectors could formulate favorable policies and incentive measures to encourage enterprises to supply more foods with nutrition claims, ultimately contributing to enhancing balanced nutrition and promoting healthy diets nationwide.

## Data availability statement

The original contributions presented in the study are included in the article/supplementary material, further inquiries can be directed to the corresponding authors.

## Ethics statement

The study was conducted in accordance with the Declaration of Helsinki, and approved by the Ethics Committee of Institute of Food and Nutrition Development, Ministry of Agriculture and Rural Affairs for studies involving humans. Informed consent was obtained from all subjects involved in the study.

## Author contributions

ZH: Conceptualization, Funding acquisition, Methodology, Project administration, Writing—original draft. HL: Data curation, Writing—review and editing. JH: Writing—review and editing.

## Footnotes

1
https://www.wjx.cn
